# 

**Published:** 1975

**Authors:** 


The
Bristol Medico-Chirurgical Journal
(Incorporating the Medical Journal of the South-West)
Established 1883

Vol. 90 (iii/iv) JULY/OCTOBER 1975 No. 335/6

### BRISTOL MEDICO-CHIRURGICAL JOURNAL

EDITOR: D. S. REEVES, M.B., M.R.C.Path.

Published Quarterly

Annual Subscription £2 Post Free

### BRISTOL MEDICO-CHIRURGICAL SOCIETY

**President 1974/75:** Dr. J. Apley, C.B.E.

**President-elect:** D. C. Bodenham, Esq.

**Honorary Secretary:** A. J. Webb, Esq., 7 Percival Road, Bristol 8.

**Honorary Treasurer:** Dr. Frank Ross, Bristol Royal Infirmary, Bristol 2.

The Society was founded in 1874. In 1883 publication of the Journal began and has continued quarterly ever since then. During the years 1949 to 1962 it appeared under the title of "The Medical Journal of the South West".

### THE BRISTOL MEDICO-CHIRURGICAL JOURNAL

**Editorial Committee**

Editor Dr. D. S. Reeves, Southmead Hospital, Bristol.

Assistant Editor Dr. A. P. Radford

Members Dr. Rhys Davies.
Dr. M. B. Lennard.
Professor R. C. Wofinden.
Dr. D. W. Wright.
The Hon. Treasurer and Hon. Secretary of the Society.

Secretary Mr. B. P. Jones, The Library, Medical School, University Walk, Bristol BS8 1TD.

### NOTICE TO CONTRIBUTORS

The Journal is especially concerned to provide a place for the recording of medical thought, interests, and practice in and around Bristol. It therefore welcomes articles that, as well as being of immediate interest to members, will document the local and contemporary medical scene for our successors.

Original articles are invited provided that they have not been published elsewhere. References in the text should be by author's name, with year of publication in parenthesis; they should be listed at the end in alphabetical order, the name of each journal or book being given in full—not abbreviated—and the title of the article added. Illustrations should be supplied ready for reproduction. Line drawings should be in Indian ink on firm white paper or board. The author will be informed if it is found necessary to ask him to pay for the making of blocks.

The following contributions will be especially welcome; notes of forthcoming events, meetings held, degrees conferred, staff appointments, honours and public appointments and other local news.

### FINAL EXAMINATION FOR THE DEGREES OF M.B., Ch.B.
### JUNE 1975

PASS

WITH HONOURS

Terence Stanley CHADWICK—with Distinctions in Child Health, Mental Health and in Clinical Pathology.
David John DAWSON—with Distinction in Clinical Pathology.
Patricia Anne HAMILTON—with Distinctions in Child Health, Clinical Pathology and in Obstetrics and Gynaecology.
Janice Ann KOHLER—with Distinctions in Medicine, Community Health, Surgery and in Clinical Pathology.
Anne Marie LASHFORD—with Distinctions in Community Health and in Child Health.
Sarah Elizabeth O'CALLAGHAN—with Distinctions in Child Health, Mental Health and in Obstetrics and Gynaecology.
Steven Howard SACKS—with Distinction in Surgery.

Niall Andrew AITKEN
Christine ANSONS
Jane Fulton Mcllwaine ATKINSON
Winston Agnelo BARCELLOS
Penelope Ann BARTON
Mieczyslaw Jerzy BIZON
Michael BRADA
Margaret Jane BRADSHAW
Kevin Peter BROWN
Barbara Ann CADGE
Hugh Doran CALVEY
Philip Henry CHAMBERS
Robert Anthony CHAPMAN
Andrew James CHARLTON
Nicholas Martin Parry CLARKE
Paul Grant COBNER
Wendy Jacqueline COE
Cecil Bernard COLACO
Gary Arnold COOK
Hilary Susan COOLING
Andrew COULSON
Stephen Alistair COURT—with Distinctions in Surgery and in Clinical Pathology
Sheila Margaret DALE
Nancy Patricia DARROCH—with Distinction in Mental Health
Colin James DAVIES
Anne Elizabeth DAVIS
Anita Jane DONLEY—with Distinction in Clinical Pathology
Angela Margaret DOUGLAS
Edmund William DRAPER
Susan Caryl EDWARDS
Timothy John FALLON
Felix Joseph FERNANDES
David Ian FIELD
Josephine Olwen FLEMING
Alan Robert FRASER
Martin John FREEMAN
Paul GAYTON
Shamim Sadrudin Manji GILANI
Glenys Laura GOULSTONE
Evan Andrew Meredith GREVILLE
Nicholas Vivian GRIFFIN
Andrew Brian GRIFFITHS
Jennifer Susan Sleator GWOZDZ
Peter Field HALEY
William Eric HANKS
Robert Braith HARBORD
Deborah Margaret HARRISON
Martin Timothy Christopher HIGGINS
Alison Maynard HILL
Mark Edmund HOWELL
Shahina Firdos JAFFER
David Andrew JONES
Terence John KEMPLE
David Michael Lawrence KERSHAW
Pravin Kumar LAKHANI
David LEVAN
Richard James LONGMAN
Andrzej Ryszard LUKSZA
Peter George LUMB
Catherine Joyce LYNE
Catherine-Anne McMULLAN
Rosemary Elizabeth MARKS
Kerridwen MARTIN-JONES
Andrew John Dundee MILLAR
Robert Edward Wade MILLER
David MORGAN
Steven MORRIS
Brenda Mary MOSEDALE
Nigel MOUSLEY
Paul Joseph NAGLE
Frederica Erna NEWBERY
Kathleen Clare NICHOLS
Richard James PAGE
Simon John PAGE
Mark George PALAZZO
Paul Anthony PATCHETT
Arvind Chhotu PATEL
Margaret Georgina PRENTICE
Allan Paul REDGRAVE
Mair RICHARDS
John Graham RIDLEY
Geoffrey Norman SHILLAM
Howard Scott Charles SIDDALL
Ian John SIMPSON
Andrew Paul SLADE
Phillip Malcolm SMITH
Michael Harry SORENSEN
Michael John STOWER
Abdul Majid Aboobakar TAYUB
John Leslie THORN
Henry Clark UMPLEBY
Grace Miriam VERRALL
Christopher Julian WALLIS
Peter Gordon WARDLE
Suman WASON
Steven Howard WHALEN
Graham WHINCUP
James Frith WILLIAMSON
Robert Geoffrey WILLIS
Christine Elizabeth WOODS

### FACULTY OF MEDICINE
### THE DEGREE OF MASTER OF SURGERY
### JUNE 1975

DISSERTATIONS APPROVED

Emanuel Olaofe OLURIN
Oyinade OLURIN

### FACULTY OF MEDICINE
### THE DEGREE OF DOCTOR OF MEDICINE
### JUNE 1975

DISSERTATION APPROVED

Richard Keith JACOBY

### FACULTY OF MEDICINE

### THE DEGREE OF DOCTOR OF PHILOSOPHY
### JUNE 1975

DISSERTATIONS APPROVED

El Sammani EL GAILI
Rosalind Margaret GASKELL
Christine GIBBS
Graham Rex HOLLAND
Robert James HOLMES
Timothy John NEWBY

